# Public Knowledge and Perception of Drinking Water Quality and Its Health Implications: An Example from the Makueni County, South-Eastern Kenya

**DOI:** 10.3390/ijerph19084530

**Published:** 2022-04-09

**Authors:** Patrick Kirita Gevera, Kim Dowling, Peter Gikuma-Njuru, Hassina Mouri

**Affiliations:** 1 Department of Geology, University of Johannesburg, Johannesburg 2006, South Africa; kim.dowling@rmit.edu.au (K.D.); hmouri@uj.ac.za (H.M.); 2 School of Science, STEM College, RMIT University, Melbourne, VIC 3001, Australia; 3 Department of Environmental Science and Land Resources Management, South Eastern Kenya University, Kitui P.O. Box 170-90200, Kenya; pnjuru@seku.ac.ke

**Keywords:** potentially harmful elements, high fluoride, water salinity, population awareness

## Abstract

Due to the semi-arid nature of Makueni County in South-Eastern Kenya, there is a high dependence on groundwater resources for domestic use. Reliance on this source of potable water may have health implications for the population, given the presence of several naturally occurring and potentially harmful elements reported from aquifer source rocks, soil, and water in the area. A survey involving questionnaires and focus group discussions (FGDs) was conducted with 115 individuals to determine the local population’s knowledge, attitude, and perceptions of their drinking water quality and its health impacts. The results show that most respondents (67%) preferred piped water because it was pre-treated and not saline. Only 29% of the respondents were very satisfied with the taste of their drinking water, while the rest complained about varying salinity levels, ranging from slightly salty to very salty. This low satisfaction might have influenced the low daily drinking water consumption (1–2 L) by most respondents. Health issues reported by many (43%) respondents in the area include diarrhoea and gastrointestinal upsets, which may be associated with the saline nature of the drinking water. Elevated fluoride (F^−^) in the local groundwater was reported, and the health effects remain a concern. Although 91% knew someone with dental fluorosis, 53% did not know the deleterious effects of high F^−^ in drinking water. Most respondents (59%) associated the salty nature of the water with dental fluorosis, and as a result, 48% avoided drinking the salty water to prevent the condition. Despite the high prevalence and known psycho-social effects, most people did not perceive dental fluorosis as a severe health threat. The increased health risks associated with high salinity and high F^−^ in drinking water in Makueni County are poorly understood by most residents, regardless of their education, gender, or age. This warrants an immediate public health education programme and detailed epidemiological studies to determine all the health effects associated with naturally occurring, potentially harmful elements in groundwater in the area.

## 1. Introduction

The availability of clean and reliable potable water is critical for people’s wellbeing, especially in arid and semi-arid regions. In recent years, compounding factors, including climate change, rapid population growth, urbanisation, and ever-increasing industrialisation, have led to over-exploitation of the already constrained water resources [1,2]. Local governments have limited capacity in most sub-Saharan Africa to meet the increasing water demands of their population, and consumers have resorted to using unregulated water resources to meet their needs [3]. The use of unprotected wells and dams as well as untreated river water is common in regions lacking reliable water supplies. This practice, however, puts people’s health at risk and exposes the population to potentially harmful substances, including naturally occurring potentially harmful elements (NOPHE). These include high salinity and often occur with elevated levels of other toxic and non-toxic elements such as iron (Fe), fluoride (F^−^), and selenium (Se) [4,5,6].

Groundwater ecosystems are essential to humans and the global economy because they act as a source of drinking water, and wide industrial and agricultural use. However, factors including the over abstraction, industrialisation, and uncontrolled waste deposition have put quality and quantity strains on this important resource [7,8]. Additionally, the presence of NOPHE in an area can compromise the quality of groundwater resources. Therefore, understanding groundwater ecosystems in regions that highly rely on this resource is crucial in order to maximise its domestic and industrial benefits and reduce potential deleterious health implications [7].

Globally, governments in arid regions face the challenge of providing safe and reliable drinking water to their populations. However, the trust and perception of the consumers regarding the quality of their drinking water affects their satisfaction and use of the water sources [9]. This trust is usually influenced by factors such as the organoleptic properties of the water (mostly clarity and taste) and previous health issues linked to the water sources [9]. In Pakistan, a public knowledge survey on drinking water quality showed that in areas where people did not like their drinking water quality and taste, most were also aware that the drinking water affected their health [10]. Despite reported health complications from using the available water sources, most respondents of the study did not treat their drinking water or report issues to the authority [10]. Conversely, in northern South Africa, nearly two thirds of participants of a questionnaire survey did not associate drinking water with potentially causing any health complications despite the water having bacterial contamination as well as being unpalatable [11]. Lack of safe drinking water practices has been reported in communities using contaminated water sources. For example, in rural southern India, some participants of a FGD reported that they warm or filter their drinking water and only boil it for children or sick adults [12].

In the semi-arid Makueni County in South-Eastern Kenya, the public water supply by local governments and non-governmental organisations (NGOs) include piped water from nearby springs, rivers and dams, as well as groundwater sources [6,13]. Government water supplies often cover the population living in urban areas, leaving those in rural areas to rely on unprotected and under-regulated water sources, with a high reliance on groundwater sources. If the groundwater is affected by NOPHE, it is often difficult for the population to discern safe and unsafe water sources [5,13,14,15]. This is a critical issue, given that the local population is uninformed about safe drinking water practices. Such cases lead to poor hygiene practices, including incorrect drinking water treatment methods.

Makueni County falls under an arid and semi-arid region with unreliable surface and rainwater [6]. The government has implemented measures, such as improving piped water supply, drilling public boreholes, shallow wells and constructing earth dams, to improve the water supply [16]. However, about 64% of the local population still use unimproved water sources for domestic and agricultural purposes [17,18]. Groundwater sources in Makueni, as well as in neighbouring counties, have been reported to contain several NOPHE, such as F^−^ and Fe, and concerning parameters, such as salinity in concentrations above the recommended limits in drinking water [5,6,19,20]. This has resulted in health complications, such as dental fluorosis and the reported undesirable taste of the drinking water, leading to the abandonment of some boreholes in the region [5,6,21].

Despite these water quality issues reported in Makueni County and other parts of the world, little is known on the level of knowledge that the affected population has. For example, in South-Eastern Kenya, only one study has been conducted in the neighbouring Machakos County to determine the local population’s perceptions and understanding of drinking water quality [21]. The study reported that about 80% of parents with children affected by dental fluorosis thought drinking salty water caused the disease. Similarly, in the Kenyan Rift Valley, Moturi et al. [22] found that most people did not associate dental fluorosis with high F^−^ in groundwater, nor did they understand proper preventive measures. Despite several studies showing the occurrence of potentially harmful elements in drinking water in Kenya [5,6,21], limited research has addressed community and individual perceptions on drinking water quality and their understanding of measures to mitigate adverse health effects [21,22]. Therefore, analysis is relevant in Makueni County, which warrants the current study.

The effectiveness of any strategy to solve health problems can be determined by the value assigned to the health threat by the affected community [23]. This statement asserts that it is difficult to prevent or reduce the health effects of NOPHE in drinking water if the affected populations are not aware of the risk factors associated with such elements. Additionally, the community’s perception of any disease and the severity of its impact necessitates the urgency of mitigation. However, most non-communicable diseases and conditions are rarely given more than cursory attention in public health education programmes, due to the slow and long-term manifestation of symptoms and illnesses [24]. Unfortunately, this lack of public’s knowledge of such diseases has been shown to increase the contraction and severity of their impacts [25,26]. In contrast, a high level of community knowledge of the risks of endemic diseases results in improved prevention and mitigation [24,27].

In addition to the effects of water quality on human health, the amount of water consumed influences health. Consumption of water below the recommended intake levels may cause dehydration and has been related metabolic and functional health complications [4,28,29,30]. Factors such as individuals’ age, gender, physical activity, and body size, as well as environmental conditions, such as temperature, influence daily water intake levels [4]. No study has reported the amount of water consumed by the local population in Makueni County. Therefore, it has not been established as to whether the local population has adequate water intake and whether this can have health implications.

Drinking-water sources in the county, which include boreholes, springs, shallow wells and tap water, have been reported to contain elevated concentrations of F^−^ and salinity higher than the recommended values in up to 50% of the analysed water sources [5,6,19,31]. A recent study in the area [6] reported evidence of the effects of the high F^−^ and salinity concentrations with cases of dental fluorosis and complaints of undesirable salty water from some boreholes by the local population. 

These findings prompted the current study, which is aimed to investigate the public knowledge and perception of drinking water quality, focusing on F^−^ and salinity, and options for water treatment available to the southern region of Makueni County. For the first time in Makueni County, we report the general population’s perception of their drinking water quality across different ages and occupations. This work is significant because, to mitigate the negative health implications of the identified NOPHE in drinking water, the level of public knowledge and attitudes on the issue must be established and acted upon accordingly. Additionally, the survey enables to determine the amount of water consumed by the population and considers possible consequent health implications. This study also demonstrates how natural ecosystems, such as groundwater resources in Makueni County, benefit the local society who highly depend on it, while also raising awareness of the potential health implications associated with these resources and ways to conserve them [32,33].

## 2. Study Area

### 2.1. Location and Population

**Figure 1:**
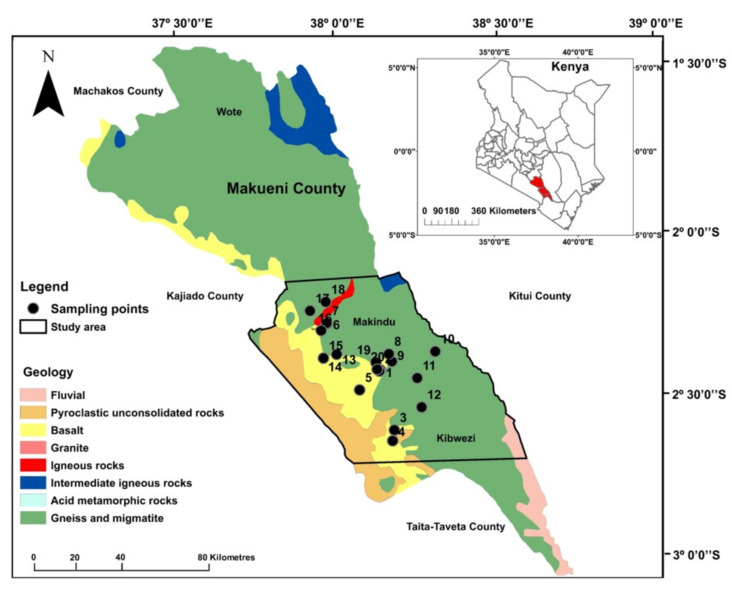
Map of Makueni County showing the location in Kenya and geology of the study area (bounded in black line). The selected households for the interviews were close to the water sampling locations (numbered 1–20) reported by Gevera et al. [6]. Figure adapted from Gevera et al. [6].

Makueni County is located in South-Eastern Kenya and borders Kitui, Kajiando, Machakos, and Taita Taveta Counties to the east, west, north, and south, respectively (Figure 1). The region is dominantly rural, except for Wote, Makindu and Kibwezi centres, which are the most significant rural towns. The population in the Makindu–Kibwezi sub-county region, where this study was carried out, is approximately 277,000, with a population density of between 63 and 100 persons/km^2^ [34]. Most of the population engages in small-scale farming, producing food crops including maize (*Zea mays*), beans (*Phaseolus vulgaris*), green grams (*Vigna radiata*), mangoes (*Mangifera indica*) and cowpeas (*Vigna unguiculata*), as well as vegetables, such as kale (*Brassica oleracea* var. *sabellica*), cowpea leaves (*Vigna unguiculata* L.), and cabbage (*Brassica oleracea* var. *capitata*).

### 2.2. Geology and Water Supply

The geology of the area includes metamorphic rocks of the Precambrian Mozambique Mobile Belt and Pleistocene–Recent volcanics [35], as shown in Figure 1. The dominant lithologies include biotite and granitoid gneisses, basalts, and pyroclastic rocks, which may act as a source of potentially harmful parameters/elements, including F^−^ and Fe [5,31]. These parameters/elements dissolve in groundwater systems and impact drinking water quality in the area [5,6,19]. Most of the population in Makueni County relies on unimproved and unprotected water sources, including springs, rivers, dams and unprotected open wells and boreholes [17,18]. The local government and non-governmental organisations (NGOs) have implemented measures, such as increasing the piped-water network, drilling community boreholes and shallow wells, and using water tankers to deliver improved quality water and supply in the county. Government water supply is primarily free or comes with a small fee used to maintain the water kiosks, while semi-private water companies charge their customers monthly.

## 3. Materials and Methods

### 3.1. Survey Methods

The survey was conducted in January 2020 within the Makindu–Kibwezi area, in the southern part of Makueni County, covering an area of approximately 2500 km^2^ (bounded in black lines in Figure 1). The results of the recent drinking water analysis in the selected area revealed F^−^ values ranging between 0.6 and 8.2 mg·dm^–3^ and salinity values between 336 and 4424 mg·dm^–3^, both of which exceed the recommended guidelines in drinking water in more than 50% of the sampled water sources [6]. Before conducting the interviews, ethical approval was obtained from the ethics committee at the University of Johannesburg (where the research was conducted) and the National Commission for Science and Technology (NACOSTI) in Kenya (where the study area located). The questionnaire included open- and closed-form questions administered to individuals, households, and one local high school. Some of the questionnaire participants were later included in the FGDs.

### 3.2. Participants and Eligibility

The households and individuals targeted for the interviews were close to drinking water sources recently sampled for physico-chemical analysis [6]. Opportunistic sampling was employed. Households that were easily accessible were approached, and members were requested to participate in the survey after receiving a briefing about the survey’s objectives. One member from the consenting households was then asked to fill the questionnaire. No family had more than one member filling the questionnaires. In market centres and towns, individuals were randomly approached to participate in the survey. A total of 82 people agreed to participate in the interviews.

One boarding high school in Makindu town was also included in the questionnaire survey, where 33 students from the junior-most and senior-most classes (aged between 13 and 17 years) participated with appropriate consent. Initially, all the students (*n* = 100) from the two classes were briefed about the project by a teacher from the school. After the briefing, 33 students volunteered to fill the questionnaire. The school (with a total population of 600 students) was included in the survey to investigate the knowledge difference between the young and old members of the populations. For this study, the term ‘students’ categorises all participants who were in the participating school as well as others from households, whilst ‘non-students’ indicates all participants who were not studying at the time of the survey. The inclusion criteria for the questionnaire interviews and FGD specified any population members in the Makindu–Kibwezi area who had lived in the area for more than three years. The respondents’ minimum age was set at 13, the minimum age of high school students in Kenya.

### 3.3. Sample Size Calculation

The estimated sample size for a study in the two sub-counties where the study area is located, Kibwezi and Makindu sub-counties, was then calculated. The population of the two counties is about 277,000 according to the Kenya National Bureau of Statistics [17]. It should be noted that the exact population size of the study area could not be established since the area falls under the two sub-counties and was not bounded by administration boundaries. The calculation of the sample size for the population in the two sub-counties used the Godden [36] sample size formula:

S = Z^2^ × p × (1 − p)/M^2^

where:

S = sample size for infinite population

Z = Z score (which is 1.812 for the 93% confidence level)

p = population proportion (which is 0.5)

M = margin of error (5%)

Therefore S = 328.3344.

Then we adjusted the sample size to the required population (that is the population of Makindu–Kibwezi sub-counties = 277,000).

Adjusted sample size = (S)/1 + [(S − 1)/population of the area]

The sample size is, therefore, 328, which is the suggested sample size for the population of Makindu and Kibwezi sub-counties. Since the study area is smaller than the two sub-counties, the population size and, therefore, sample size, should be smaller than those of the two sub-counties. The survey gathered qualitative data with descriptive and Likert scale responses of the perception and opinions of the participants and as such, a sample size of sample size of 115 is appropriate and significant. Additionally, most of the interviews were conducted in the households where one member, who represents the household, filled the questionnaire. The average household size of the area was five. Therefore, the perceptions of entire households were represented in the sample size of 115. Similar and lesser sample sizes are reported in numerous community-based health perceptions studies [10,37] and the data revealed are substantial.

### 3.4. Study Design

The interview questionnaire (Supplementary Material File S1) was synthesised from several validated tools [24,38,39]. It comprised 45 questions grouped into different categories. The first group of questions inquired about the participant's demographic information, including age, gender, occupation, marital status, home language, home village/location, household size, and income. The next questions addressed dietary habits, such as available drinking water sources, distance to these water sources, amount of water drunk daily, sources and types of food consumed, and where they were derived. Then the participants’ knowledge and perception of their drinking water quality were investigated. These questions include knowledge of drinking water contaminants present in the area, how to identify them, and how to decontaminate/remove them in drinking water. They were then asked to state their preference for groundwater or surface water sources, level of trust and satisfaction on their drinking water sources and quality, and whether they had ever been taught about safe drinking water practices.

Subsequent questions focused on knowledge about F^−^ and salinity in drinking water. These included awareness about what fluoride is, its health benefits and detriments and their knowledge on the presence, severity, causes, and preventive measures of dental fluorosis in the area. Similarly, their opinion on drinking water taste, its satisfaction, and if the taste affects their health were asked. Questions about the water's colour and smell were included to investigate reports of high Fe in groundwater in the Makueni County that causes staining of pipes as reported in literature [5]. Finally, the participants were asked if they had reported issues of water taste, colour or smell to the authorities and whether any action had been taken.

Additionally, two FGDs, each involving 10 discussants, were conducted to strengthen the analysis and improve the capacity to provide contextualised information [40]. The discussions were held in the two major towns in the area, Makindu and Kibwezi, to get people’s opinions from these areas. All the discussants had completed the questionnaires and consented to participate in the follow-up discussions. They were all adults and known to each other. This criterion was used to enable openness amongst the participants during the discussions since they were no strangers to one other.

The discussion guide was structured to investigate areas in the questionnaire that may provide more profound qualitative answers. There, the following four key questions were considered during the discussions with the participants in the survey: (i) Why do some people prefer piped water to borehole water? (ii) To their knowledge, what are the health implications arising from drinking salty water in the area? (iii) What is the prevalence of dental fluorosis, and the perception and attitudes of the general population towards people with dental fluorosis? (iv) In their opinion, what improvements need to be made regarding water quality and availability in the area? 

### 3.5. Study Procedure

In the household and school settings for the questionnaire survey, the principal investigator and the teacher involved in the survey were present when the participants were filling the questionnaires in case queries arose. The FGDs took place in quiet public settings, where all the participants were comfortable. The discussants were encouraged to express their opinions fully and freely, and the discussions were conducted in the national language, Kiswahili. The discussions lasted for a maximum of 30 min each. The principal investigator moderated the discussions, using the structured discussion questions mentioned above, and took notes after each question to capture all views. In both the questionnaire and FGDs, all respondents were literate. Still, the principal investigator had to verbally translate some questions from English to Kiswahili to ensure clarity for a few older respondents.

### 3.6. Data Analysis

The closed-form question data were transferred to Microsoft Excel 365. They were then analysed using IBM SPSS Statistics 25 to determine statistical grouping and correlation. A Chi-square test for association and Cramer’s V coefficient were used to determine the significance and strength of correlation, respectively, in some groups at a 95% confidence level. The Cramer’s V, used in larger than 2 × 2 tables, shows little correlation when values are close to 0 and strong correlations when values are close to 1. The association can be weak to moderately weak at 0–0.3, moderate to moderately strong at 0.3–0.6 and strong to very strong at 0.6–1 [41]. ArcGIS 10.5 software and Microsoft PowerPoint were used for graphical processing.

## 4. Results

### 4.1. Demographic Characteristics

About 90% of the households and 60% of individuals who were approached agreed to participate in the survey. Most individuals who declined to participate cited reasons including lack of time and no interest, due to lack of monetary compensation. A total of 115 respondents (76 males and 39 females) completed the questionnaires, and 20 participated in the FGD. This was the maximum possible number of participants the survey attained during the one-month long survey. The one-month period was within the financial and time limits available to conduct the survey in Kenya. With one exception, all the questions were fully and easily answered by all participants. A total of 30.43% (*n* = 35) of the participants did not answer the question about the amount of monthly family income.

The mean and median values were the same for most of the variable (including household size, source of drinking water, distance to drinking water, amount of daily drinking water consumption, trust, perception, and satisfaction in drinking water sources, knowledge of drinking water quality, knowledge of F^−^ and salinity health effects), indicating that the dataset was evenly distributed. Participants’ ages ranged from 13 to 62 years, with the largest group being in the 31–50-year bracket (34%). In terms of their occupation, most of the respondents were students (34%), with the second dominant category being small-scale traders (29%), while farmers were the third-largest group (10%). The rest of the respondents were carpenters, electricians, mechanics, public transporters and casual labourers, herein referred to as ‘others’. The average household size was five members. Many of the respondents (39%) had lived in the area for more than 20 years, 32% had lived for 11–20 years and 29% for less than 10 years. Only seven (6%) respondents, who were students in the boarding school, came from outside Makueni County but were included in the study because they came from neighbouring counties (Machakos and Kitui Counties) with similar geology and water quality characteristics to those of Makueni County. All respondents spoke Kiswahili and Kikamba as their first languages and English as the second.

### 4.2. Drinking Water Accessibility

**Figure 2:**
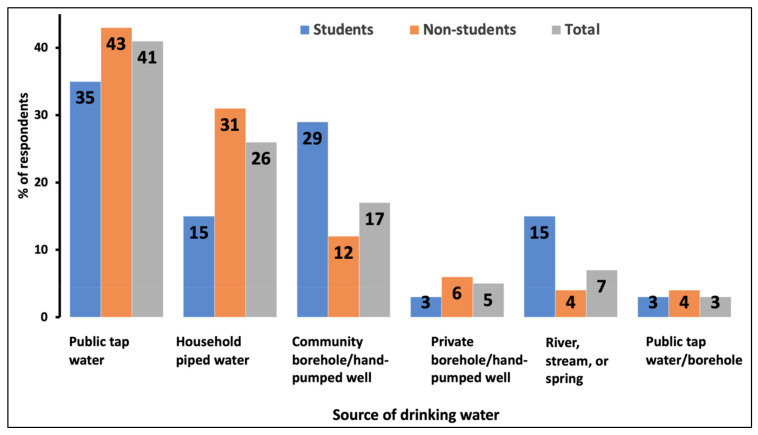
The percentage of respondents and their use of drinking water sources in the Makindu–Kibwezi area of Makueni County.

(i) Drinking water sources: Many respondents (41%) used public piped water. The second largest group (26%) used household piped water (Figure 2). Piped water is sourced from a natural spring in the Makindu area, thus having a groundwater source. Community and private boreholes and hand-pumped wells were relied on by 17% and 5% of the respondents, respectively. Other respondents relied on rivers, streams, springs, or a combination of the above sources (Figure 2). 

**Figure 3:**
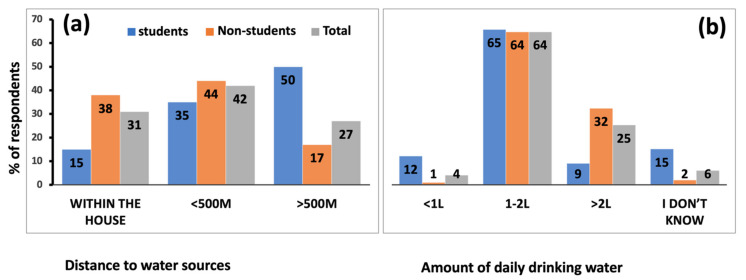
The percentage of respondents and the distance to their drinking water sources (**a**) and the amount of daily drinking water (**b**) in the Makindu–Kibwezi area of Makueni County.

A crosstab correlation shows a moderately strong (Cramer’s V = 0.53) significant (*p* = 0.005) association between the respondents’ drinking water source and occupation. Public tap water was mainly (38.3%) consumed by traders, who mostly lived in the urban centres, while students consumed mostly (55%) community boreholes and hand-pumped well water. Farmers, mostly residing in the villages, and individuals from other occupations were the primary users of private boreholes and hand-pumped well water (83.3%). There was a moderately weak (Cramer’s V = 0.27) significant (*p* = 0.02) association between the drinking water source and home area. Public tap water and household piped water were mainly (53.2%) used by participants from the Makindu and Kibwezi towns. Water sources from community boreholes and hand-pumped wells were mostly (35%) used in the eastern and southern parts of Kibwezi, which has a rural setting. Residents from the Makindu area used 80.3% of all private boreholes and hand-pumped wells water sources. There was a moderately strong (Cramer’s V = 0.46) non-significant (*p* = 0.46) correlation between the drinking water source and household income.

(ii) Drinking water availability and consumption: Only 31% of the respondents had access to a water supply within their houses, 42% had it within 500 m of their homes, while 27% had to travel for more than 500 m to obtain water (Figure 3a). A correlation to determine if the area where the participants live (rural or town) or income affects their access to drinking water was run. There was a moderately weak (Cramer’s V = 0.29) non-significant (*p* = 0.62) correlation between distance to water and areas where the participants lived. Similarly, the participants’ household monthly income did not affect the distance to their water sources, as shown by a weak (Cramer’s V = 0.19) non-significant correlation (*p* = 0.65). 

Most respondents (64%) estimated that they consume between 1 and 2 L of water per day, followed by 25% who consume >2 L per day, and 4% who consumed <1 L per day, while 6% could not quantify their daily water consumption (Figure 3b). There was a moderate (Cramer’s V = 0.42) significant (*p* = 0.00) correlation between age and amount of water consumption. There were more students (12% compared to 1% of non-students) among those who consumed <1 L water per day, while there were more non-students (32% compared to 9% of students) among those who consumed >2 L per day. Similarly, more students (15% compared to 2% of non-students) were amongst participants who did not know how much water they drank per day. The amount of water consumed was not affected by the distance to the water source, as shown by a moderate (Cramer’s V = 0.46) non-significant (*p* = 0.28) correlation.

### 4.3. Trust and Satisfaction of Drinking Water Sources

Many (47%) of the respondents indicated that they trusted their drinking water sources, 30% did not trust theirs, while 23% were not sure. This trend was not affected by age, as shown by the weak (Cramer’s V = 0.15) non-significant (*p* = 0.27) correlation between the participants’ trust and satisfaction and age. Similarly, the source of drinking water did not influence the respondents’ trust and satisfaction, as shown by a moderate (Cramer’s V = 0.37) non-significant (*p* = 0.1) correlation. Additionally, trust and satisfaction did not affect the daily drinking water consumption, as shown by the moderately weak (Cramer’s V = 0.24) non-significant (*p* = 0.57) correlation between the two.

**Figure 4:**
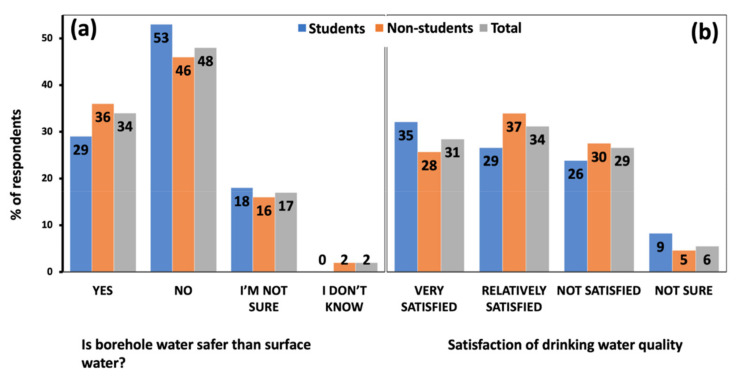
The percentage of respondents indicating their trust on the safety (**a**) and satisfaction of the quality (**b**) of their drinking water.

Only 34% of the respondents perceived borehole or shallow well water as being safer than surface water, with 48% expressing a contrary opinion, while the rest were either not sure or did not know (Figure 4a). The age of the participants and drinking water source did not influence this perception, as shown by their weak (Cramer’s V = 0.18, 0.25) non-significant (*p* = 0.47, 0.1) correlations, respectively. Although many respondents (34%) were relatively satisfied with their drinking water quality, a great diversity of opinions was reported from very satisfied (31%) to not satisfied (29%) (Figure 4b). There was a weak (Cramer’s V = 0.11) non-significant (*p* = 0.72) correlation between age and satisfaction of drinking water quality.

### 4.4. Knowledge and Perception of Drinking Water Quality

**Figure 5:**
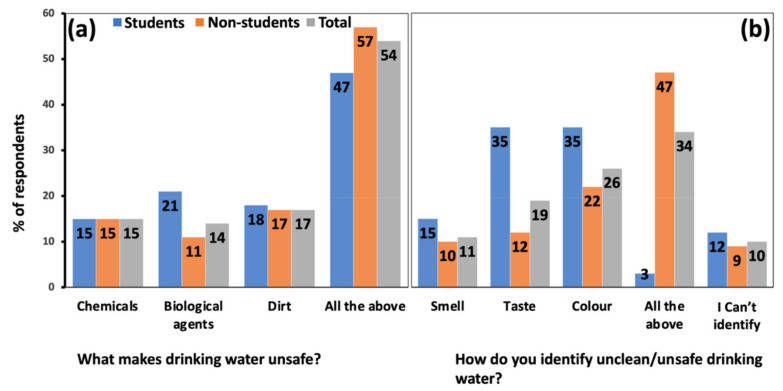
The percentage of respondents indicating their knowledge on what makes drinking water unsafe (**a**) and how to identify it (**b**).

(i) Knowledge: More than half of the respondents (54%) were aware that biological agents, chemicals, and dirt might render water unsafe for consumption (Figure 5a). The rest of the respondents reported that only one of the three agents could make water unsafe for consumption. There were weak to moderately weak (Cramer’s V = 0.13, 0.27), non-significant (*p* = 0.58, 0.48) correlations between knowledge of water contaminants and the participants’ age and occupation.

Most respondents (56%) identified water as unclean by one of the following characteristics: smell, taste or colour. The median group identified colour, 34% could identify by combining the three characteristics, and only 10% could not identify unclean water using those properties (Figure 5b). There was a moderate (Cramer’s V = 0.44) statistically significant (*p* = 0.00) correlation between age and how participants identified unclean water. Many non-students (47%), compared to students (3%), identified unclean water using all the variables. There was also a moderately strong (Cramer’s V = 0.48) statistically significant (*p* = 0.00) correlation between occupation and how participants identified unclean water. Traders were the dominant group (39%) that identified unclean water with all the three variables, while the opinions varied among other groups.

(ii) Drinking water colour and smell: Most respondents (71%) reported no colour in their drinking water, a finding that was similar in both students and non-students. Others reported drinking water with brown, black, and grey colours, particularly during the rainy season and water harvested from shallow wells. Most respondents (78%) reported no smell in their drinking water, a finding similar in both students and non-students. Those who reported smell in water associated it with old pipe systems and water that had stayed in storage tanks for an extended time. Most respondents (57%) reported that smell and colour do not affect the amount of water they consume. However, 32% reported that unpleasant smell and muddy colour affects how much water they drink, while 11% reported that water colour sometimes affects the amount of water they consume. The number of respondents who reported a smell in drinking water was similar in both students and non-students.

Some respondents (25%) had previously made complaints to local water utility companies and public health officers about unpleasant water colour and smell. However, often, these authorities took no action to address their complaints. The few responses included providing alternative water sources and installing new pipes by the local water utility, primarily to address complaints about brown colour in water.

(iii) Drinking water F^−^: when asked if they knew what fluoride is, most respondents (65%) said they knew what it is. The age of the participants did not affect their knowledge of what fluoride is, as shown by a weak (Cramer’s V = 0.153) non-significant (*p* = 0.1) correlation. Similarly, the area where participants lived, their monthly household income, and occupation did not influence their knowledge of fluoride, as shown by their respective weak non-significant correlations (Cramer’s V = 0.22, 0.22, and 0.19) (*p* = 0.36, 0.39, and 0.27).

On determining their knowledge on the benefits of F^−^ in water, only 23% of respondents knew that in the correct dose, F^−^ is beneficial for the strengthening and protection of teeth and bones (Table 1); 35% thought it is used as a disinfectant to kill germs in drinking water, and 41% did not know any benefits of F^−^ in drinking water. Many students (44%) believed F^−^ was used as a disinfectant, while among non-students, many (47%) did not know any benefits of F^−^. There was, however, no statistical correlation between age and knowledge of health benefits of F^−^ as shown by the moderately weak (Cramer’s V = 0.22) non-significant (*p* = 0.14) correlation. Similarly, the participants’ occupation did not influence their knowledge of F^−^ benefits (Cramer’s V = 0.36, *p* = 0.95).

More than half of the respondents (51%) did not know any deleterious effects of F^−^, and more non-students (57%), compared to students (38%), were in this group (Table 1). About 37% of the respondents knew that F^−^ causes dental and skeletal fluorosis; more students (50%) than non-students (31%) were in this category. A total of 12% of the participants thought F^−^ either promotes germs in water or does not have negative effects (Table 1). There was a moderately weak (Cramer’s V = 0.29) significant (*p* = 0.02) correlation between age and knowledge on the deleterious effects of F^−^. However, the occupation did not have a statistically significant correlation with knowledge on the deleterious effects of F^−^ (Cramer’s V = 0.35, *p* = 0.11).

Most of the respondents (95%) knew someone with stained teeth (dental fluorosis) and the majority (54%) estimated the number of people they knew with stained teeth as ranging from ‘quite a lot’ to ’a lot’ (Table 1). There was a weak (Cramer’s V = 0.19) non-significant (*p* = 0.6) correlation between the area of residence and the number of people with dental fluorosis that the participants knew. Despite most respondents knowing someone with stained teeth, most (59%) thought the staining is caused by salty water, and only 21% identified F^−^ as the causative agent (Table 1). Others did not know the cause of the staining (17%) or thought it was caused by a lack of proper dental hygiene (3%). Both students and non-students had a similar view of the causes of dental fluorosis. The home area and occupation of the participants did not influence the knowledge of what causes teeth staining, as shown by moderate and non-significant correlations (Cramer’s V = 0.37, *p* = 0.72; Cramer’s V = 0.3, *p* = 0.6), respectively.

Due to the popular belief that salty water is the cause of teeth staining in the area, the most common preventive measure is avoiding drinking salty water, as reported by many respondents (48%) (Table 1). Only 14% reported avoidance of high F^−^ drinking water as the preventive measure, 12% proposed improving dental hygiene, and 17% did not know any preventive measure (Table 1). There was a moderate (Cramer’s V = 0.34) significant (*p* = 0.01) correlation between age and knowledge about dental fluorosis preventive measures. There were slightly more non-students (51%) than students (41%) among those who reported avoiding salty water, as well as those who suggested the avoidance of high F^−^ drinking water (15% non-students and 12% students). There were more students (26 %) than non-students (6%) among those who suggested improvement of dental hygiene as the preventive method of dental fluorosis.

(iv) Drinking water salinity: Most respondents (76%) reported drinking water to have a salty taste. The taste ranged from slightly salty, as reported by most of the respondents (56%) to salty (15%) and very salty (5%) (Table 1). Respondents expressed different levels of satisfaction with the taste of their drinking water. Many (35%) were relatively satisfied, followed by 29% who were very satisfied, whilst 22% were dissatisfied (Table 1). The age of the participants did not influence their level of drinking water satisfaction (Cramer’s V = 0.25, *p* = 0.62).

Due to the varying salinity level, most respondents (64%) reported that the drinking water taste affects how much water they drink. More non-students (70%) than students (50%) reported that the drinking water taste affected how much water they drank. Conversely, more students (50%) than non-students (29%) indicated that taste does not affect how much water they drink. Therefore, salinity influenced how much water some of the adult participants drank compared to students. This was shown by a weak (Cramer’s V = 0.19) significant (*p* = 0.03) correlation between age and how taste affected how much water the participants drank in the area. 

The association between the participants’ description of water taste and if the taste affected how much water they drank is supported by a moderately strong (Cramer’s V = 0.5) significant (*p* = 0.02) correlation. Most participants (51.2%) reported that drinking water taste did not affect how much water they drank and categorised their drinking water as fresh. Similarly, most (63.5%) of those whose drinking water taste affected the amount of water they drank categorised their water as slightly salty. Many (45%) respondents reported no health complications from their drinking water, while 43% associated salty water with diarrhoea, gastrointestinal upsets and even tonsillitis. Other health complications related to salty water by the participants include biological vectors for pathogens and dental fluorosis.

(v) Water treatment: Most respondents (55%) reported that they did not treat their drinking water before consumption. This number was higher among students (68%) than non-students (49%). There was a moderate (Cramer’s V = 0.31) significant (*p* = 0.02) correlation between occupation and whether participants treated their drinking water. Participants with the minor occupations (carpenters, electricians, mechanics, public transporters, and casual labourers) were the dominant group (37%) among those who treated their drinking water, followed by farmers (27%). Students (41%) were the dominant group among those who did not treat their drinking water. Household monthly income did not influence whether participants treated their drinking water or not (Cremer’s V = 0.167, *p* = 0.67).

The main treatment methods reported by the participants include boiling (43%) and the use of chlorine-based disinfectants (14%). The reason for using these two methods was to remove biological contaminants. The rest of the respondents used both of these two methods as well as water filters. About 9% of the respondents reported having suffered from amoebiasis and thus, always boiled their drinking water before consumption. Most respondents (79%) reported being taught about water quality by a school instructor, public health officer, the media, local water utility and the church.

## 5. Discussion

### 5.1. Drinking Water Accessibility

Most (67%) people in the Makindu–Kibwezi area rely on public piped water or groundwater resources (public and private boreholes and shallow wells) for domestic uses. The government and a private water utility supply piped water from a freshwater spring located in the Makindu area [6]. Therefore, the main drinking water sources in the area have a groundwater source, whose quality is highly influenced by the local geology [6]. Of these water sources, trust in piped water was higher (48%) than groundwater (34%). From the focus group discussions, the opinion that piped water was reliable was particularly common among all the discussants from Kibwezi town, who complained of dry wells during the dry seasons. In addition, participants stated that the piped water was pre-treated and, therefore, safer, fresh (not saline) and had a pure and natural taste. Similar opinions were reported by a study in Brazil where some participants preferred tap water because it was pre-filtered [42]. The discussants who preferred borehole water in the current study perceived it as being safer due to its natural source, which they associated with less anthropogenic contamination and being rich in natural “minerals”. All the discussants who preferred borehole water had personal boreholes or wells at their homesteads. Although most respondents had easy access to drinking water sources, nearly a third (27%) of the local population had to travel more than 500 m to access domestic water. This indicates that a significant amount of the population in the southern region of Makueni County faces challenges in accessing drinking water.

### 5.2. Drinking Water Quality and Satisfaction

The level of knowledge and perception of a community towards potential health implications associated with their drinking water is reflected in their practices [25]. Most (>70%) participants were satisfied with their drinking water colour and smell, except for cases of brown, black, or grey colour (silt) reported during the rainy seasons or caused by old pipes. Although many (47%) respondents trusted their drinking water sources, only a third were very satisfied with the water taste. This trend was contrary to a study in Pakistan, where people who did not like their drinking water taste, similarly disliked those water sources [10]. During interviews, it was also noted that most people were reluctant to find issues with their borehole water for the fear that, if reported, they would have their water source shut down by the government. This might explain the difference between the high number of those who trusted their drinking water and the low number of those satisfied with the taste. This low level of satisfaction can be associated with varying salinity levels in drinking water in the area.

Salinity: High salinity present in drinking water has an important influence on the amount of water consumed by the residents. Up to 63% of the participants who described their drinking water as slightly salty indicated that this taste caused some limitation to their daily water consumption. As a result, participants in the FGDs reported that most families living in areas with high saline water in Kibwezi are forced to buy bottled water from shops or water vendors and use the saline water for cooking and other domestic purposes. Salinity values ranging between 336 and 4424 mg·dm^–3^ have been reported in more than 50% of water sources in Makueni County [5,6,19,31], which were characterised as being poor to unacceptable [6]. In addition to affecting drinking water taste, prolonged consumption of high saline water can have several health implications, including diarrhoea, hypertension, and eclampsia in pregnant women [43,44,45,46]. Cases of diarrhoea and gastrointestinal upsets experienced by children and people who recently moved to the area were reported during the FGDs. Similar health complications were also associated with drinking highly saline water in Bangladesh [47]. Despite reporting on such health complications, many (45%) respondents did not associate the consumption of saline water with any significant health implications, apart from the undesirable taste. Similar results were reported in the coastal regions of Bangladesh [47] and northern South Africa [11], where participants did not associate any health implications to drinking water with high salinity (Bangladesh) and high levels of vectors (South Africa).

According to Gevera et al. [6], the major cations and anions in the drinking water in the area are Na, Ca, Mg, Cl^−^ and SO_4_^2−^ . High dietary intake of Na, Ca, and Cl^−^ have been associated with cases of hypertension, congestive heart failure and asthma, and drinking water with high Mg levels has been reported to have a laxative effect [48,49,50,51,52]. Similarly, high NaCl salts in water have been associated with gastrointestinal complications [48]. These elevated levels may be linked to several health issues reported in the area. High blood pressure was reported as the fifth major health risk factor in Makueni County in 2016 [53]. Although high blood pressure is mainly linked to the rise of a sedentary lifestyle in Kenya [53], a recent study showed that the consumption of high saline groundwater could also contribute to rising rates of hypertension [46].

Fluoride: Although most (65%) of the respondents reported knowing the word fluoride, only a few (23%) knew its dietary benefits as well as its deleterious effects (37%). This indicates a low level of knowledge of the effects of fluoride ingestion and its health implications, despite being present in higher than the recommended levels (1.5 mg·dm^–3^) in 50% of drinking water sources in the area [6,19]. Both students and non-students had an insufficient level of knowledge. However, more non-students (57%), compared to students, (38%) did not know the deleterious effects of F^−^. The impact of high F^−^ concentrations in drinking water were apparent during the interviews, with most (91%) of the respondents knowing at least one person with dental fluorosis. Unlike in Kibwezi town, participants of the FGDs in Makindu town reported that they were not concerned by the aesthetic appearance of people with dental fluorosis and did not see the condition as a health threat. This may be due to the higher prevalence of the disease in Makindu than in Kibwezi as evidenced by some discussants in Kibwezi town who described dental fluorosis as “*a disease that mainly affects people in Makindu area*” and that “*you can identify people from Makindu because most have stained teeth*”. Similar studies, such as in Palestine [25], have shown that people are tolerant of the appearance of dental fluorosis in high-prevalence areas. In contrast, in areas with low dental fluorosis prevalence, people were more concerned with the affected individuals’ dental appearance [54].

Despite most participants indicating that they were not concerned by dental fluorosis, a study in northern Iran showed that the condition can cause serious complications, including difficulty in chewing and even mobility challenges and osteoporosis due to structural damage and deterioration of the bones [55]. These may be risk factors for the local population in Makueni County, given the high F^−^ in the area’s drinking water sources [6,19]. Additionally, several studies have shown that dental fluorosis has significant psycho-social effects on an individuals’ wellbeing [56,57,58,59]. For example, teenagers with dental fluorosis are reportedly concerned by their dental appearance, which affects their social and academic life [58,59]. This was evident during the FGDs when discussants from Kibwezi town reported that people find those with dental fluorosis as socially undesirable. Therefore, the condition should be considered significant because of its physical, psychological, and social impacts.

Amount of drinking water consumed: Estimating total water intake requires the summation of drinking water, water in beverages, and food moisture intake. Direct water intake is a significant measure to maintain good health and optimal body function because drinking water and fluids contribute to approximately 80% of the total water intake [4,28,60]. Additionally, we note that water requirements vary and depend on human physiology, gender, occupation, and life stage, among other factors [4,28]. Therefore, inadequate water intake is significant in promoting health for all individuals in any community. Consuming an insufficient amount of water is associated with an increased risk of dehydration, fatigue, high blood pressure, kidney stones, and poor cognitive performance [4,61,62]. The recommended daily total water intake ranges between 1.3 and 3.3 L for children below 18 years and 2.3 and 3.7 L for adults [4,28,30,63]. Similarly, the World Health Organisation (WHO) guideline values for chemical contaminants are given with an assumption that an adult consumes 2 L of water per day, which is equivalent to 3 L from all fluids and foods [63]. It should be noted that the respondents’ total dietary habits were not surveyed during the interviews, and only drinking water was considered.

Despite the complexity of estimating the amount of water intake, the ingestion of drinking water in Makueni County is generally low. Being a semi-arid tropical region, most (64%) of the respondents estimated that they drank about 1–2 L of water daily and less than a third (25%) drank >2 L daily. Additionally, water salinity influenced the amount of water most participants drank. Participants who described their water as salty reported that taste affected how much water they drank. This could be caused by the fact that some discussants noted that salty water did not quench their thirst, resulting in most people drinking less water than they desired.

Makueni County is a dominantly rural area with a semi-arid climatic condition, where non-mechanised farming and other physically intense activities are common. People living in such conditions might face dehydration and related complications if they consume less than the recommended amount of drinking water. This might affect the health and physical activities of people in the area. Although this study did not consider other water intake (dietary and beverages) sources, we recommend an increase in drinking water intake by the population of Makueni County to avoid the health complications that might arise due to their low drinking water intake. Due to the unpalatability of some drinking water sources in the area, which might affect water consumption, the people are recommended to increase fruits and vegetable intake, which increases their total water intake. Overall, an education campaign is recommended to ensure hydration and minimise its short- and long-term effects.

### 5.3. Public Knowledge of Safe Drinking Water Practices

The local population’s knowledge of water quality and health implications was poor. Despite high dental fluorosis cases in the area, most respondents were misinformed on the benefits and deleterious effects of F^−^ in drinking water as well as its mitigation measures. High saline drinking water was linked to the cause of dental fluorosis by people in the area, with the avoidance of salty water used as a preventive measure. For example, one participant in the FGD indicated that they knew that teeth staining was “*caused by a lot of minerals in the water*” and therefore associated the ‘salinit*y*’ with the ‘minerals’ in the water. A similar perception was reported in Machakos County, adjacent to Makueni, where 80% of parents with children affected by dental fluorosis reported salty water as the condition’s cause [21]. 

These results show that, despite the high concentrations of F^−^ in drinking water in the area and the high prevalence of dental fluorosis, the local population is not well informed on the health impacts of F^−^ in their diet. This lack of awareness was slightly higher in non-students than students. Similarly, general knowledge of safe drinking water practices was lacking among the participants. For example, only about half of the participants knew that the combination of physical, biological, and chemical agents could affect water potability. Non-students seemed to be more knowledgeable in this regard, even though students are expected to be taught about hygiene in school.

Due to the semi-aridity nature of the area, the main concern of most respondents and discussants in the FGDs was the availability of reliable water sources for domestic and agriculture and not the quality. On learning about the health implications associated with their drinking water during our interviews, all the respondents expressed interest in learning more about drinking water quality, the impacts of poor water quality and better prevention measures. There is, therefore, a need for the local authorities to implement public education programmes in the area to educate people about water hygiene and the presence of NOPHE in the area that can potentially affect their health. Preventive measures, including defluorination and the provision of fresh drinking water, are encouraged in the area.

The groundwater ecosystem provides (supply of domestic and agricultural water) and supports (supply of nutrients) services to the local populations of the area [7,8]; this study shows that understanding these resources in Makueni County is crucial in maximising their benefits while reducing their potential health implications. This is achieved through educating the population on the presence of potentially harmful elements in their water systems and implementing defluorination and desalination programmes while involving the local population.

The key assumptions of this study are that all participants gave correct answers to the interview questions. An important limitation of the study might include some answers given by the participants which are not necessarily correct but desirable to them. For example, most participants feared that their water sources might be closed if found with potentially harmful elements and therefore, they might have indicated being satisfied with their water sources and taste. Additionally, the cases of diarrhoea and gastrointestinal upset reported by participants was assumed to be only caused by drinking highly saline water. However, unhygienic conditions and bacterial contamination could also result in such cases. Future work could target a larger or more diverse sample population to better investigate and reduce any sampling or statistical errors.

## 6. Conclusions

This paper reports on the practices, knowledge, and perceptions of the local population in the Makindu–Kibwezi area of Makueni County on their drinking water quality and its health implications. The results show that many people trust piped water compared to borehole/shallow well water due to its year-long reliability and low salinity, compared to the latter. Due to the high salinity in drinking water in the area, most people are not satisfied with their drinking water taste, which highly negatively influences their daily water consumption. The high salinity in drinking water was linked to several health complications, such as diarrhoea and gastrointestinal upsets, especially in children and people who recently relocated to the area. Notably, most respondents, especially students, were inadequately informed about the different water pollutants and how to identify unclean water. Additionally, most participants were poorly informed and uninformed about F^−^ benefits and deleterious effects in their drinking water. This is despite F^−^ being present in higher than recommended values in about 50% of their drinking water sources and nearly all the respondents knowing someone with dental fluorosis. This dental condition was only perceived as an aesthetic issue, especially in the high-prevalence Makindu area. Most people had poor practices on F^−^ and salinity preventive measures, as most used boiling and chlorine-based disinfectants, which only eliminate biological pollutants.

Based on these findings, this study recommends several measures to be implemented by the local and governments or international organisations, such as the World Health Organisation, to improve the local population’s health status. The low awareness of dental fluorosis as a health threat and the known psycho-social implications of the condition needs to be addressed through tailored educational programmes. Furthermore, a detailed epidemiological study on the occurrence of the aforementioned diseases and others associated with high salinity and F^−^ in drinking water in the area should be undertaken. Further studies should also compare the occurrence of these diseases within the different population demographics, such as age, gender, and occupation, in order to assess the level of effects. Due to the participants' potentially low daily drinking water intake, studies are needed to determine hydration status in Makueni County and areas with similar climatic, groundwater quality, and drinking water patterns. The impacts of limited water supply in semi-arid regions, the concentration of undesirable and potentially harmful elements in the available drinking water, and a limited understanding of the health effects of drinking high F^−^ and saline water require greater investigation and education of the population. Furthermore, by noting the benefits of groundwater ecosystem services in Makueni County as well as its potential health implications, this study is intended to raise the interest of local policy makers to look for ways to make drinking water safe for human consumption. This study specifically addresses ecosystem services, which links the landscape with the provision of human necessities and explores the relationship between natural assets, ecosystems, and human well-being.

## Figures and Tables

**Table 1:** Public perception on the benefits, effects, and preventive measures linked to F^−^ content and salinity in drinking water in the Makueni County.

		Students % (*n*)	Non-Students % (*n*)	Total % (*n*)
Do you know what fluoride is?	Yes	76 (26)	60 (49)	65 (75)
	No	24 (8)	40 (32)	35 (40)
What are the health benefits of fluoride?	Kill germs	44 (15)	31 (25)	35 (40)
	Protect & strengthen teeth and bones	29 (10)	20 (16)	23 (26)
	I don’t know	26 (9)	47 (38)	41 (47)
	None	0 (0)	2 (2)	2 (2)
What are the negative effects of fluoride?	Promotes germs in water	0 (0)	9 (70	6 (7)
	Dental fluorosis	50 (17)	31 (25)	37 (42)
	I don’t know	38 (13)	57 (46)	51 (59)
	None	12 (4)	4 (3)	6 (7)
How many people with stained teeth do you know?	Very few	15 (5)	12 (10)	13 (15)
	Few	24 (8)	28 (23)	27 (31)
	Quite a lot	24 (8)	35 (28)	31 (36)
	A lot	38 (13)	17 (14)	23 (27)
	None	0 (0)	7 (6)	5 (6)
What causes the teeth staining?	Salty water	53 (18)	62 (50)	59 (68)
	Lack of proper dental hygiene	9 (3)	1 (1)	3 (4)
	Excess fluoride in water	21 (7)	21 (17)	21 (24)
	I don’t know	18 (6)	16 (13)	17 (19)
How do you prevent teeth staining?	Avoid salty water	41 (14)	51 (41)	48 (55)
	Improve dental hygiene	26 (9)	6 (5)	12 (14)
	Avoid high F drinking water	12 (4)	15 (12)	14 (16)
	I don’t know	21 (7)	16 (13)	17 (20)
	Either of the above options	0 (0)	12 (10)	9 (10)
Describe your drinking water taste	Slightly salty	56 (19)	56 (45)	56 (64)
	Salty	12 (4)	16 (13)	15 (17)
	Very salty	12 (4)	2 (2)	5 (6)
	Fresh	21 (7)	26 (21)	24 (28)
How satisfied are you with your drinking water taste?	Very satisfied	26 (9)	30 (24)	29 (33)
	Relatively satisfied	38 (13)	33 (27)	35 (40)
	Relatively dissatisfied	3 (1)	20 (16)	15 (17)
	Dissatisfied	32 (11)	17 (14)	22 (25)

## Data Availability

The datasets generated and/or analysed during the current study are not publicly available to maintain the anonymity of the participants of the research but are available from the corresponding author on reasonable request.

## Supplementary Materials

The following are available online at https://www.mdpi.com/article/10.3390/ijerph19084530/s1, File S1: Information leaflet, question sheet and informed consent form.
