# Supplementary material for: Public Knowledge and Perception of Drinking Water Quality and Its Health Implications: An Example from the Makueni County, South-Eastern Kenya
Source: Int J Environ Res Public Health. 2022 Apr 9;19(8):4530. doi: 10.3390/ijerph19084530 (PMC9032672; doi:10.3390/ijerph19084530)
Supplement: Supplementary file 1 [file ijerph-19-04530-s001.zip › ijerph-1614171-supplementary.pdf]

Information leaflet, question sheet and informed consent form.

**STUDY TITLE: Naturally occurring potentially harmful elements in the Makueni County environment, South-Eastern Kenya: health implications and community awareness**

INVESTIGATOR: Patrick Kirita Gevera

INSTITUTION: The University of Johannesburg, Auckland Park Kingsway

Campus TELEPHONE NUMBERS: +27 847514248
E-MAIL: pattygevera@gmail.com

ETHICAL APPROVAL: The study had been approved by the University of Johannesburg and the ethics clearance for this study has been granted by the ethics committee of the University of Johannesburg.

To the Participant: *The principal investigator, is available to answer any questions or address issues that you do not understand regarding this study. You may take home an unsigned copy of this consent form to think about or discuss with family or friends before making your decision to participate in this study. Your participation is purely voluntary*

Participant Number: _______________

PROSPECTIVE PARTICIPANT INFORMATION LEAFLET

Naturally occurring potentially harmful elements in the Makueni County environment, South-Eastern Kenya: health implications and community awareness

This is an invitation to participate in a research project in the Geology Department at the University of Johannesburg, South Africa. Please take a moment to read through this information sheet, as the details of how you can assist us are explained. Participation in the project is entirely voluntary. This means that we need your permission to interview you. You can however choose not to participate. You are free to withdraw from the project at any time.

What are our aims with this project? The aim of this study is to determine the concentrations of naturally occurring potentially harmful elements in rocks, soil, drinking water and food crops in selected areas of Makueni County in order to determine their health implications. We would like to interview you, to determine if you know of any health implications associated with these elements on the local population in the area and how well the population are aware of the exposure and prevention measures to these elements.

What do we ask from you? All we ask from you is to participate in our discussion or fill the questionnaires provided to the best of your knowledge. The questions will involve your knowledge of health implications associated with named elements affecting members of the community and how to prevent exposure to these potentially harmful elements.

What are the benefits of participation in this study? By participating in this survey, you will provide information on health implications caused by potentially harmful elements in drinking water and food in Makueni County and how well you are aware of their exposure and prevention. Based on the results, we can give suggestions to the local government on ways to minimize or prevent exposure to these potentially harmful elements and educate you on such ways.

Are there any risks involved? There are no risks involved in this interview. All we need is your time to answer the questions which are based on your daily observations and knowledge.

Will everybody know everything about you? Not at all. Any information you provide will be confidential, collected anonymously, and cannot be traced back to you.

If you have any questions: If you have any questions or inquiries about this researcher, you can contact the researcher **Mr. Patrick Gevera** at pattygevera@gmail.com or +27 847514248

If you agree to participate in the study, please read and sign the attached consent form. Thank you for your kind consideration.

Patrick Kirita Gevera
Department of Geology,
University of Johannesburg

LETTER OF INFORMED CONSENT FORM

To whom it may concern

The purpose of this form is to inform you about this study, which is being carried out in your community, and to seek your consent to take part in it.

Project title: **Naturally occurring potentially harmful elements in the Makueni County environment, South-Eastern Kenya: health implications and community awareness**

This study involves asking you questions about the occurrence of health implications that can be caused by potentially harmful elements in drinking water and food in Makueni County and how to prevent them. Your assistance in answering the questions is requested.

Confidentiality and anonymity will be maintained throughout the time. Should you wish to stop with the interview, you can do so at any time.

If this letter is clear to you, and you agree to take part, then write your name and sign below to indicate your informed consent.

I,____(Full names and Surname), the undersigned, have read the Prospective Participant Information Sheet, that whatever doubts I initially had, have been cleared and voluntarily agree to be a part of this study, which aims to determine possible health effects caused by naturally occurring potentially harmful elements in drinking water and food crops in Makueni County. I understand that my participation is completely voluntary and that the results will be used purely for academic purposes. I can withdraw from this study at any time if I wish and I have sufficient opportunity to ask questions. Of my own free will, I hereby agree to participate in the interviews or completion of questionnaires relating to this study. I also hereby state that I understand that there is no monetary reward for my involvement in these interviews.

_______________________ _______________________

Signature of Participant Date (dd-mm-yy)
(**for participants below 18 years, Parents/guardians should sing here**)

_______________________ _______________________

Signature of Researcher Date (dd-mm-yy)

PROSPECTIVE PARTICIPANT QUESTION SHEET

Naturally occurring potentially harmful elements in the Makueni County environment, South-Eastern Kenya: health implications and community awareness

Name of Investigator: ……………………… Starting Time:……………………

Date:…………………………………… Ending Time:…………………….

Investigator Introduction:

Hello, my name is Patrick Kirita Gevera, the principal investigator of this study. I would like to ask you some questions about the quality of drinking water and food in your area (Makueni County). May I have this opportunity to speak to you?

All information stated in this questionnaire will be kept strictly confidential. Please be entirely honest and answer all questions where applicable. It is NOT NECESSARY to state your name on this sheet. All information will be kept anonymous.

1. General information

**Demographic Questions**	**Answers**
1. What is your age?	
2. What is your gender?	
3. What is your occupation?	
4. What is your marital status?	
5. What is your home language?	
6. Which village/location do you live in?	

7. How long have you lived in your village/location?	
8. How many members are there in your household?	
9. What is the monthly household income?	1. <10,000 Ksh.<br>2. 10,001-25,000 Ksh.<br>3. 25,001-50,000 Ksh.<br>4. 50,001-100,000 Ksh.<br>5. >100,000 Ksh.

**Additional information:**

2. Dietary habits

Questions	Answers
1. What are the available drinking water sources in your neighborhood/village?	1. Public tap water<br>2. Household piped water supply<br>3. Community borehole/hand pump<br>4. Private borehole/hand pump<br>5. Others (please specify)
2. What is the source of drinking and cooking water at home?	1. Public tap water<br>2. Household piped water supply<br>3. Community borehole/hand pump<br>4. Private borehole/hand pump<br>5. Others (please specify)
3. How far is your household water source?	1. Within the house<br>2. Less than 50 meters<br>3. 51-100 meters<br>4. 101-500 meters<br>5. More than 500 meters
4. How much water do you drink per day?	1. Less than 1 liter<br>2. 1 to 1.5 liters<br>3. 1.5 to 2 liters<br>4. More than 2 liters<br>5. I don't know
3. What is the usual source of food consumed in your household?	1. Own farm<br>2. Neighborhood farms<br>3. Farmers market<br>4. Supermarket/ shops<br>5. Others (please specify)
5. What vegetables do you normally eat?	1. Kale (*sukumawiki*)<br>2. Cabbage<br>3. Cow peas leaves (*kunde*)<br>4. Others (please specify)
6. What cereals do you normally eat?	1. Maize<br>2. Rice<br>3. Grain legumes (beans, cow peas, pigeon peas, green grams)

	4. Others (please specify)
7. What other foods do you usually eat?	

3. **Knowledge and perception about drinking water quality**

**Questions**	**Answers**
**Part (i) (general water use)**	
1. Do you trust the sources of your drinking water to be safe for your health?	1. Yes<br>2. No<br>3. Not sure
2. How satisfied are you with the quality of your drinking water?	1. Very satisfied<br>2. Relatively satisfied<br>3. Not satisfied<br>4. Not sure
3. What can make drinking water unsafe for consumption?	1. Biological agents<br>2. Chemicals<br>3. Dirt (soil)<br>4. All of the above<br>5. Others (please specify)
4. How can you identify unclean/unsafe water?	1. Smell<br>2. Taste<br>3. Color<br>4. I cannot identify<br>5. Others (please specify)
5. Do you treat your water before drinking or cooking with it?	1. Yes<br>2. No
6. What are you doing to improve drinking water quality?	1. Water neutralization<br>2. Boil<br>3. I use filters<br>4. Nothing<br>5. Others (please specify)
7. Do you think borehole/well water is safer for drinking and cooking than tap or stream/river water?	1. Yes<br>2. No<br>3. Relatively<br>4. Not sure<br>5. I don't know
8. Have you ever been taught/informed about drinking water safety? By whom?	Yes/ No<br><br>1. In school<br>2. Local water utility<br>3. Public health officers<br>4. Media<br>5. Others (please specify)
9. Do you wish to be informed about drinking safety?	1. Yes<br>2. No<br>3. Maybe

	4. Not important
**Part (ii) (fluoride focused questions)**	
10. Do you know what fluoride is?	1. Yes<br>2. No
11. What are the benefits of fluoride in drinking water?	1. To kill germs<br>2. To protect and strengthen teeth and bones<br>3. I don't know<br>4. Others (please specify)
12. What are the bad effects of fluoride in drinking water?	1. Promote germs in water<br>2. Causes dental fluorosis (brown teeth)<br>3. Causes skeletal fluorosis (bending of bones and pain in joints)<br>4. I don't know<br>5. Doesn't have bad effects
13. Do you know anyone around your home with stained/brown teeth?	1. Yes<br>2. No
14. How many are they? Few, quite a few, quite a lot, a lot.	1. Very few<br>2. Few<br>3. Quite a lot<br>4. A lot
15. What causes the teeth staining?	1. Salty water<br>2. Lack of proper dental hygiene<br>3. Excess fluoride in water<br>4. I don't know<br>5. Others (please specify)
16. How do you prevent the teeth staining?	1. Avoid salty water<br>2. Improve dental hygiene<br>3. Avoid high fluoride drinking water<br>4. I don't know<br>5. Others (please specify)
**Part (iii) (salinity focused questions)**	
17. Does your drinking water have a taste?	1. Yes<br>2. No
18. If yes, how can you describe the taste?	1. Slightly salty<br>2. Salty<br>3. Very salty<br>4. Extremely salty<br>5. Other (please specify)
19. How satisfied are you with the taste of your drinking water?	1. Very satisfied<br>2. Relatively satisfied<br>3. Relatively dissatisfied<br>4. Dissatisfied
20. Does the taste of your water affect how much you drink?	1. Yes<br>2. No
21. Does the quality of your drinking water affect your health in anyway?	1. Yes<br>2. No<br>3. Maybe

	4. I don't know
22. If yes, please explain how	
**Part (iv) (iron focused questions)**	
23. Does your drinking water have color?	1. Yes<br>2. No
24. If yes, how can you describe the color?	1. Brown<br>2. Black<br>3. Grey<br>4. Other (please explain)
25. If yes, when do you notice the color?	1. When it rains (seasonal)<br>2. Always once in a while<br>3. Other (please specify)
26. Does your drinking water have a smell?	1. Yes<br>2. No
27. How can you describe the smell?	1. Unpleasant<br>2. Pleasant<br>3. Other (please specify)
28. Does the color and/or smell affect how much water you drink?	1. Yes<br>2. No<br>3. Sometimes
29. Have you made a complaint about the water taste/smell/ color before?	1. Yes<br>2. No
30. To whom did you complain?	1. Local water utility<br>2. Public health officers<br>3. Others (please specify)
31. What was the result?	1. Action was taken immediately<br>2. Action was taken after some time<br>3. No action was taken<br>4. Other (please specify)
32. If action was taken, please specify?	1. Filters were installed in public water systems/homes<br>2. New pipes were installed<br>3. Alternative water sources were introduced<br>4. Other (please specify)

For use by research personnel:

Participant

number_________________________

Date: ______________________
